# Cardioautonomic lability assessed by heart rate variability changes in Royal Canadian Mounted Police cadets during the cadet training program

**DOI:** 10.3389/fpsyg.2023.1144783

**Published:** 2023-09-27

**Authors:** Taylor A. Teckchandani, J. Patrick Neary, Katie L. Andrews, Kirby Q. Maguire, Laleh Jamshidi, Jolan Nisbet, Robyn E. Shields, Tracie O. Afifi, Shannon Sauer-Zavala, Lisa M. Lix, Rachel L. Krakauer, Gordon J. G. Asmundson, Gregory P. Krätzig, R. Nicholas Carleton

**Affiliations:** ^1^Canadian Institute for Public Safety Research and Treatment-Institut Canadien de Recherche et de Traitement en Sécurité Publique (CIPSRT-ICRTSP), University of Regina, Regina, SK, Canada; ^2^Faculty of Kinesiology & Health Studies, University of Regina, Regina, SK, Canada; ^3^Anxiety and Illness Behaviours Lab, Department of Psychology, University of Regina, Regina, SK, Canada; ^4^Department of Community Health Sciences, University of Manitoba, Winnipeg, MB, Canada; ^5^Department of Psychology, University of Kentucky, Lexington, KY, United States; ^6^Department of Psychology, University of Regina, Regina, SK, Canada

**Keywords:** heart rate control, generalized anxiety disorder (GAD), cardiac, RCMP cadets, cardioautonomic dysfunction

## Abstract

**Objective:**

The current study examined variations in cardioautonomic lability during the Royal Canadian Mounted Police (RCMP) Cadet Training Program (CTP) between cadets starting their training who did or did not screen positive for one or more mental health disorders (i.e., posttraumatic stress disorder [PTSD], major depressive disorder [MDD], social anxiety disorder [SAD], generalized anxiety disorder [GAD], panic disorder [PD], alcohol use disorder [AUD]).

**Methods:**

Electrocardiogram (ECG) signals integrated into Hexoskin garments were used to record ECG and heart rate Over the 26-week CTP. There were 31 heart rate variability (HRV) parameters calculated using Kubios Premium HRV analysis software. Mann–Whitney *U*-tests were used to perform groupwise comparisons of participant raw values and HRV during the CTP.

**Results:**

A total of 157 cadets (79% male) were screened for any mental disorder using self-report surveys and then grouped by positive and negative screening. Analyses indicated a statistically significant (*p* < 0.05) decrease in low frequency (LF): High Frequency (HF) variability during CTP, but only for cadets who endorsed clinically significant anxiety symptoms on the GAD-7 at the start of their training. There were no other statistically significant groupwise differences.

**Conclusion:**

The results indicate the participants have excellent cardiac health overall and suggest potentially important differences between groups, such that cadets who endorsed clinically significant anxiety symptoms on the GAD-7 showed less variability in the LF:HF ratio over the course of the CTP. The relatively lower variability suggests decreased parasympathetic tone in those without clinically significant anxiety symptoms. The results also have important implications for future investigations of cardioautonomic dysfunction and chronic hypothalamic pituitary adrenal (HPA) axis deviations in policing populations with anxiety disorders; specifically, cardioautonomic inflexibility related to cardiovascular morbidity and mortality. In any case, the current results provide an important baseline for future cardiac research with cadets and serving officers.

## 1. Introduction

Correctional workers, firefighters, paramedics, municipal and provincial police, public safety communicators, and the Royal Canadian Mounted Police (RCMP) are all examples of public safety personnel (PSP) who work to keep Canadians safe (Canadian Institute for Public Safety Research and Treatment, 2021). PSP are vocationally required to frequently engage with extremely stressful situations (Carleton et al., 2019). Stressors that involve real or threatened exposure to death, significant injury, or sexual assault can be described as potentially psychologically traumatic events (PPTEs) (Canadian Institute for Public Safety Research and Treatment, 2021). Increased symptoms of mental health disorders, such as posttraumatic stress disorder (PTSD), major depressive disorder (MDD), generalized anxiety disorder (GAD), panic disorder (PD), social anxiety disorder (SAD), and alcohol use disorder (AUD), have frequently been linked to PPTE exposures (Carleton et al., 2019), and are collectively referred to as posttraumatic stress injuries (PTSI; Public Safety Canada, 2019). Approximately half (i.e., 50.2%) of serving RCMP officers screened positive for one or more PTSI (Carleton et al., 2018), which is approximately five times higher than the prevalence (i.e., ≈ 10%) in the Canadian general population (Statistics Canada, 2012). Mental health disorders have been associated with poor health, including declines in cardiovascular health [e.g., coronary heart disease, hypertension, diminished heart rate variability (HRV); Tully et al., 2013; Pelletier et al., 2017]. Earlier PTSI identification and intervention could improve the mental and physical health of RCMP and other PSP.

The regulation of heart rate is a complex process involving interplay between neuroendocrine and neuroautonomic systems. Neurohormonal cascades occur between the neuroendocrine hypothalamic pituitary adrenal (HPA) axis and neuroautonomic vagus nerve-mediated parasympathetic suppression of the sinoatrial node (SA node). The regulation process produces beat-to-beat adjustments in cardiac inotropy and chronotropy, and sustained variations in myocardial performance, which respond to environmental demands or stressors (Roth et al., 2012; Faes et al., 2013; Tafet and Nemeroff, 2020). The magnitude and frequency of vagus parasympathetic nerves system (PNS) efferents innervating the sinoatrial node are regulated by the interplay between gamma-aminobutyric acid (GABAergic) sympathoinhibitory pathways from the caudal ventrolateral medulla acting upon the parasympathetic outflow tracts within the rostral ventrolateral medulla (Barman and Yates, 2017).

Neurohormonal pathways have been associated with symptoms of mental health disorders (Edwards and Guilliams, 2010; Tafet and Nemeroff, 2020). The HPA axis is responsible for altering immune, metabolic, and neuroautonomic processes in an intricate negative feedback loop to facilitate acute and prolonged adaptations to stress (Dishman et al., 2000; Faes et al., 2013; Wood, 2014). Pituitary release of adrenocorticotropic hormone (ACTH) in response to an acute external stressor triggers the activation of the “fight or flight” response system, regulated by the noradrenergic neurons in the locus coeruleus/norepinephrine system in the brain (Roth et al., 2012). The processes modulate stress response fidelity and efficacy, which means acute and chronic exposure to stressors can precipitate HPA axis dysfunction, facilitating a neuroendocrine cascade that negatively impacts end organ function over time (Verma et al., 2010; Flandreau et al., 2012; Roth et al., 2012; Wood, 2014; Tafet and Nemeroff, 2020).

Assessing susceptibility to changes in cardioautonomic lability requires examining the situational factors that contribute to a negative adaptation, including PPTE exposures. Stressor-induced HPA axis dysfunction can vary greatly in clinical presentation, but acute overactivation or prolonged exposure to stressors that precipitate activation of the negative feedback system can result in acute HPA axis hypersensitivity or chronic hyposensitivity (Flandreau et al., 2012; Roth et al., 2012). Previous studies evidenced physiological consequences for immune, metabolic, and cardioautonomic health in clinical populations, and HPA axis deviations have been observed to substantially contribute to physiological impairments in persons with anxiety, panic, or mood disorders (Kemp et al., 2012; Alvares et al., 2013; Tully et al., 2013; Chalmers et al., 2014; Bilgin et al., 2015; Dimitriev et al., 2016).

HPA axis dysfunction or sustained deviations of the HPA axis away from a basal state can lead to cardioautonomic inflexibility or decreased autonomic lability during stressor exposures (Roth et al., 2012; Barbieri et al., 2017) such as exposure to a PPTE. Decreased autonomic lability with prolonged HPA axis deviations caused by acute or chronic environmental stressors appears observable through evaluating the temporal and stochastic dynamics of changes in heart rate (Hagerman et al., 1996; Task Force of the European Society of Cardiology and the North American Society of Pacing and Electrophysiology, 1996; Jain and Tiwari, 2014; Schmalenberger et al., 2019; Tolin et al., 2021). HRV analyses have been widely popularized as useful and easy tools for evaluating the effects of changes in autonomic lability in those with acute or chronic stressors (Tully et al., 2013; Levine et al., 2016); however, there are considerable methodological challenges and concerns related to meaningful use of HRV analyses for assessing stress and mental health (Billman, 2013; Hayano and Yuda, 2019; Vila et al., 2019).

The RCMP Study (Carleton et al., 2022) provides a unique opportunity to address several gaps in the extant literature regarding mental health and cardioautonomic lability among cadets and serving RCMP. The current study was designed to examine differences in cardioautonomic lability during the 26-week RCMP Cadet Training Program (CTP) by comparing participating cadets who screened positive for one or more mental health disorders (i.e., PTSD, MDD, GAD, PD, SAD, AUD) when starting their training to cadets who did not. Observing potential differences and changes in cardioautonomic lability among RCMP cadets provides a basis for building tools to identify physiological characteristics or precursors to PTSI. The current study was also designed to provide baseline measures of cardioautonomic lability for future analyses as we follow RCMP cadets over the course of their careers. For the current study, RCMP cadets who screened positive for any mental health disorder at T1 (i.e., pre-training) were hypothesized to have lower HRV than cadets who did not screen positive (Gorman and Sloan, 2000; Miu et al., 2009; Pittig et al., 2013; Chalmers et al., 2016; Levine et al., 2016; Shinba, 2017; Spangler et al., 2021).

## 2. Materials and methods

### 2.1. Procedure

The current study is part of the wider RCMP Study being conducted at RCMP Depot in Regina, Canada. Full details on the RCMP study can be found in the protocol paper (Carleton et al., 2022). The RCMP Study was approved by the University of Regina Institutional Research Ethics Board (File No. 2019–055) and the RCMP Research Ethics Board (file No. SKM_C30818021312580). The RCMP Study was also approved through a Privacy Impact Assessment as part of the overall approval by the National Administrative Records Management System (NARMS) (file No. 201611123286) and Public Services Procurement Canada (PSPC) (file No. 201701491/M7594174191).

### 2.2. Data and sample

Participants were RCMP cadets (*n* = 157; 79% male) starting the 26-week CTP. Inclusion criteria included cadets who were Canadian citizens or permanent residents, 19 to 57 years old, and who could fluently read, write, and speak either English or French. Cadets must also have met several recruiting requirements, including security clearances, medical examinations, a polygraph test, and minimum physical standards (Hembroff et al., 2020). There were no conditions requiring exclusion of persons otherwise qualified for the CTP.

### 2.3. Self-report measures

Mental health disorders symptoms were assessed using web-delivery of the self-report survey at pre-training, which included the PTSD Check List 5 (PCL-5; Weathers et al., 2013a); the 9-item Patient Health Questionnaire (PHQ-9; Kroenke et al., 2001); the Panic Disorders Symptoms Severity Scale, Self-Report (PDSS-SR; Shear et al., 1997); the 7-item Generalized Anxiety Disorder scale (GAD-7; Spitzer et al., 2006); the Social Interaction Phobia Scale (SIPS; Carleton et al., 2009); and the Alcohol Use Disorders Identification Test (AUDIT; Saunders et al., 1993). For the PCL-5, per the Diagnostic and Statistical Manual of Mental Disorders, 5th edition (DSM-5; Vahia, 2013), participants reported on their lifetime exposures (i.e., prior to attending the CTP) to a specific list of PPTEs provided by the Life Events Checklist for the DSM-5-Extended (LEC-5) (Weathers et al., 2013a). The LEC-5 does not include “sudden and unexpected death of someone close to you” as a potential index PPTE, making the screening process arguably more conservative (Ashbaugh et al., 2016). Participants select an index PPTE (i.e., “Consider which event from the list was the worst, most distressing event. If more than one of these events happened to you, select the one event that currently causes you the most distress”) against which to rate their past month symptoms using the PCL-5 items. A positive screen on the PCL-5 required participants to report exposure to at least one LEC-5 item, meet minimum DSM-5 criteria for each PTSD symptom cluster subscale (e.g., intrusions, avoidance, negative alterations in cognitions and mood, and alterations in arousal and reactivity), and exceed the clinical cut off of >32 (Weathers et al., 2013b).

PHQ-9 symptoms were reported for the previous 14 days, PDSS-SR symptoms for the previous 7 days, GAD-7 symptoms for the previous 14 days, SIPS symptoms for no specified time frame, and AUDIT symptoms for the last year. Based on published guidelines for total scores, positive screenings for each scale were established: PHQ-9 > 9 (Vahia, 2013); PDSS-SR > 7 (Houck et al., 2002); GAD-7 > 9 (Swinson, 2006); SIPS >20 (Carleton et al., 2009); and AUDIT >15 (Gache et al., 2005). Instead of being validated as definitive diagnostic tools, all measures have been validated for screening to identify people who may need more therapeutic care.

### 2.4. Sociodemographic variables

Sociodemographic characteristics, including sex (i.e., male, female), age (i.e., 19–29 years, 30–39 years, 40–49 years, 50–59 years), marital status (i.e., single, separated/divorced, married/common-law), province of residence [i.e., Western Canada (British Columbia, Alberta, Saskatchewan, Manitoba), Eastern Canada (Ontario, Quebec), Atlantic Canada (Newfoundland & Labrador, Prince Edward Island, Nova Scotia, New Brunswick), or Northern Territories (Yukon, Northwest Territories, Nunavut)], and education (i.e., high school graduate or less, some post-secondary school, and university degree/4-year college or higher) were used for detailed descriptions of groupwise comparisons and covariates (Carleton et al., 2022).

### 2.5. Cardioautonomic lability measures

The RCMP Study design originally used electrocardiography (ECG) to measure HRV through analyses of successive beat-to-beat intervals of sinus origin. The successive daily ECG recordings were collected by cadets wearing Hexoskin wearable biosensor garments (Carré Technologies Inc., Montréal, Canada) that were modified for operational policing requirements. The cadets were asked to record at least one 300-s-long resting state recording per day upon waking (Task Force of the European Society of Cardiology and the North American Society of Pacing and Electrophysiology, 1996). The cadets were then encouraged to wear the garment during training tasks, with the objective of obtaining at least three to five recordings per week, per participant. Collected recordings were downloaded to a secure server for offline processing and analyses (Carleton et al., 2022).

All ECG recordings were collected using the integrated 3-lead ECG in the Hexoskin wearable biosensor garments at a frequency of 256 Hz. The ECG lead exported is equivalent to Lead I configuration. To analyze changes in HRV, the raw ECG waveforms collected by the Hexoskin wearable biosensor garments were exported as a European Data Format (EDF) file, with waveform recognition, pre-processing, and beat-to-beat analysis afforded by Kubios HRV Premium (Version 3.3.0 HRV Biosignal Analysis and Medical Imaging Group, Finland). The total number of records collected during the 26-week CTP period totaled 6,699 across 157 cadets. After preprocessing and manual review of the ECG recordings collected by the Hexoskin shirts, a total of 3,828 records were excluded from the sample due to poor signal quality over the 300 s recording period, eliminating 56 cadets (75% males). Poor signal quality was defined as a 300 s segment collected during rest that featured more than 5% artifact as detected by a moving average filter featured in Kubios Premium (Task Force of the European Society of Cardiology and the North American Society of Pacing and Electrophysiology, 1996). The remaining 2,871 records were uniformly analyzed and manually reviewed to ensure the recordings contained no ectopic activity.

HRV time domain analysis used a 50-millisecond threshold for both NNxx and pNNxx, denoting the number of successive heart beats that vary by more than 50-milliseconds. Both approximate entropy and sample entropy used an embedding dimension of two beats and a tolerance of 0.2 x standard deviation in the 300 s window. Spectral analysis used a window width of 300 s with a 50% window overlap. The low frequency (LF) band ranged from 0.04–0.15 Hz, where the high frequency (HF) band ranged from 0.15–0.4 Hz. All HRV parameters summaries were calculated by Kubios HRV Premium and processed for export as a text file to be compiled using Microsoft Excel (Microsoft Corporation, Seattle, USA) and imported to IBM SPSS Statistical Analysis Software (IBM, v.26 Premium, New York, USA) for statistical analysis.

The dependent variables were HRV analyses parameters derived from electrocardiogram data collected using the Hexoskin wearable biosensors by cadets daily during training. HRV was evaluated using a combination of 31 index, time domain, nonlinear, and power spectral density parameters. Index values included stress index, parasympathetic nervous system index, and sympathetic nervous system indexes, calculated to normalize HRV compared to normal resting values provided by Kubios Premium. Time domain parameters included analyses of the time difference between heart beats measures in milliseconds and in beats per minute. These included the average and standard deviation of the interval between heart beats (RR interval), average and standard deviation of heart rate, minimum and maximum heart rate recorded in the sample window, root mean squared standard deviation of the RR interval, the number of successive RR intervals that exceed 50 milliseconds, and the baseline width of the RR interval histogram. Nonlinear analyses included Fourier transforms to represent variability in the frequency domain. Values derived from the power spectral density function of the 300-s sample window included total, low, and high frequency power represented as milliseconds squared, as well as the percent contribution to the total power that each of the spectral bands represent. The ratio of low-to-high frequency contribution to the total spectral power was also included (LF:HF ratio). Nonlinear analyses included Poincaré analysis featuring SD1 and SD2, SD2/SD1 ratio, as well as measures of approximate entropy, and sample entropy. Detrended fluctuation analysis and recurrence plot analyses were also included to investigate changes in stochastic and harmonic properties of heart rate over the 300 s window. HRV parameters were calculated for each 300-s resting state recording, with within-participant averages calculated across the 26-week training period to produce a single value for each participant, per dependent variable. A mirrored calculation was performed to capture the standard deviation of the within participant HRV parameters to produce a single standard deviation value per participant during the 26-week CTP.

### 2.6. Statistical analyses

Descriptive statistics during pre-training included sociodemographic variables (i.e., sex, age, marital status, ethnicity, province of residence, and education). A series of *t*-tests and an analysis of variance (ANOVA) were used to assess for differences across sociodemographic groupings. The prevalence of positive screenings were computed as percentages based on self-report screening tools. Familywise error rate in multiple comparisons from *post hoc* testing was controlled using Holm-Bonferroni modifications to alpha values. Cohen’s *d* values were calculated as standardized effect sizes for *t*-tests and interpreted as small (*d* = 0.20), medium (*d* = 0.50), and large (*d* = 0.80). Partial eta squared ( $\eta_{p}^{2}$ ) were calculated as standardized effect sizes for ANOVA tests and interpreted as small ( $\eta_{p}^{2}$ = 0 . 01), medium ( $\eta_{p}^{2}$ = 0.06), and large ( $\eta_{p}^{2}$ = 0.14) (Cohen, 2013).

Groupwise comparisons were performed using Mann–Whitney *U* tests with grouping determined by GAD-7 binary screening for those who endorsed clinically significant anxiety symptoms on the GAD-7 and those who did not (i.e., positive, negative) upon the start of the CTP. Two-tailed Mann–Whitney *U* tests were used to analyze groupwise differences due to independence of observations, unequal group sizes, and violations of the assumption of normality. Shapiro–Wilke tests were used to assess data distributions of the dependent variables individually by group, with all HRV measures between groups revealing nonparametric distributions (*p* < 0.05). Effect size analyses used the biserial rank correlation coefficient. There were no statistically significant sex differences for number of recordings per participant or age.

## 3. Results

Self-reported participant sociodemographic characteristics and self-reported mental health disorder symptom scores are provided in Table 1. Participants were mostly male (79.0%), between the age of 19–29 years old (51.7%), and single (48.3%) or married/common-law (48.3%). Participants were mostly from Western Canada (55.2%; i.e., British Columbia, Alberta, Saskatchewan, Manitoba) and reported having either some post-secondary school (36.0%) or a university degree, 4-year College or higher level of education (32.0%). Self report average body weight was 188.49 ± 12.32lbs, average height was 70.78 ± 2.32 inches, and average BMI was calculated to be 26.51 ± 1.73 kg/m^2^.

**Table 1:** Median of heart rate variability parameters by group during CTP.

	Parameter	Median (IQR)		U statistic	*P*-value	Effect size (*r*)
		Negative (*n* = 95)	Positive (*n* = 12)			
	Stress Index	11.72 (4.97)	9.62 (5.58)	262	0.741	0.03
Index values	SNS Index	0.86 (1.82)	0.62 (1.14)	235	0.163	0.07
	PNS Index	−0.74 (1.45)	−0.20 (0.80)	188	0.472	0.14
Time domain	Mean RR (ms)	824.70 (141.53)	852.91 (153.45)	213	0.301	0.10
	STD RR (ms)	38.96 (26.63)	52.67 (27.02)	245	0.565	0.06
	RMSSD (ms)	30.01 (32.72)	48.16 (33.74)	201	0.227	0.12
	Mean HR (bpm)	74.72 (13.09)	72.18 (13.64)	233	0.455	0.07
	STD HR (bpm)	3.92 (1.89)	4.05 (2.31)	279	0.931	0.01
	Min HR (bpm)	64.64 (12.88)	63.36 (11.21)	244	0.556	0.06
	Max HR (bpm)	90.54 (15.19)	86.66 (15.25)	221	0.358	0.09
	NNxx	38.20 (77.82)	71.47 (71.52)	204	0.244	0.12
	pNNxx	12.69 (24.18)	21.25 (21.71)	199	0.217	0.12
	TINN (ms)	200.56 (134.56)	264.05 (128.12)	249	0.605	0.05
Spectral analysis	LF (%)	67.07 (10.62)	63.96 (13.95)	184	0.147	0.15
	HF (%)	24.85 (12.73)	28.35 (15.60)	204	0.244	0.12
	LF:HF Ratio	4.14 (2.64)	2.72 (1.62)	251	0.625	0.05
	LF (ms^2)	1224.63 (1688.69)	1702.19 (1127.88)	189	0.168	0.14
	HF (ms^2)	383.56 (1194.02)	929.45 (2748.37)	231	0.438	0.08
	Total Power (ms^2)	1594.24 (2794.74)	2843.50 (4248.12)	149	0.051	0.20
Nonlinear analyses	SD1 (ms)	21.51 (23.28)	34.37 (23.92)	230	0.429	0.08
	SD2 (ms)	73.08 (37.24)	92.56 (29.64)	223	0.373	0.09
	SD2:SD1	3.48 (1.19)	3.08 (1.08)	265	0.774	0.03
	ApEn	1.10 (0.10)	1.10 (0.08)	218	0.336	0.10
	SampEn	1.37 (0.22)	1.44 (0.16)	164	0.082	0.17
	Alpha 1	1.33 (0.25)	1.26 (0.17)	213	0.301	0.10
	Alpha 2	0.87 (0.10)	0.91 (0.15)	179	0.128	0.15
	D^2	2.47 (0.96)	3.04 (0.99)	270	0.829	0.02
	Mean line length	11.85 (2.80)	12.41 (9.26)	217	0.328	0.10
	Max line length	266.23 (138.94)	206.24 (94.92)	259	0.709	0.04
	Recurrence rate (%)	35.15 (6.04)	33.78 (8.88)	226	0.397	0.08
	Determinism (%)	98.52 (1.07)	98.13 (1.26)	277	0.908	0.01

*Results are statistically significant at the *p* < 0.05 level.

No participants screened positive for MDD, PD, AUD, SAD, or PTSD, which is consistent with expectations given the high levels of health among new cadets (Carleton et al., 2023). Small statistically significant effects for cadets that endorsed clinically significant anxiety symptoms considered a positive screen on the GAD-7 only were observed for total spectral power and sample entropy (i.e., *p* = 0.051, *r* = 0.20; *p* = 0.082, *r* = 0.17, respectively). Tables 1, 2 provide the HRV parameters by group during the CTP for cadets who screened positive for GAD using the GAD-7 questionnaire at the start of the CTP.

**Table 2:** Standard deviation of heart rate variability parameters by group during CTP.

	Parameter	Median (IQR)		U statistic	*P*-value	Effect size (*r*)
		Negative (*n* = 95)	Positive (*n* = 12)			
Index values	Stress Index	1.09 (0.52)	1.32 (3.02)	124	0.431	0.08
	SNS Index	0.86 (0.62)	1.26 (1.68)	123	0.418	0.08
	PNS Index	3.23 (0.21)	3.22 (0.33)	79	0.060	0.19
Time domain	Mean RR (ms)	3.30 (2.11)	4.56 (10.04)	88	0.102	0.16
	STD RR (ms)	91.76 (41.30)	107.94 (66.76)	90	0.113	0.16
	RMSSD (ms)	10.92 (6.57)	11.18 (14.82)	96	0.148	0.15
	Mean HR (bpm)	15.56 (12.39)	21.77 (35.35)	128	0.488	0.07
	STD HR (bpm)	8.81 (3.78)	9.10 (14.02)	150	0.860	0.02
	Min HR (bpm)	1.31 (1.45)	1.06 (2.24)	108	0.245	0.12
	Max HR (bpm)	7.83 (3.21)	9.42 (12.08)	137	0.630	0.05
	NNxx	17.73 (19.54)	27.86 (64.82)	114	0.307	0.10
	pNNxx	40.25 (31.82)	42.51 (36.04)	98	0.162	0.14
	TINN (ms)	3.10 (2.19)	3.98 (2.77)	111	0.275	0.11
Spectral analysis	LF (%)	10.64 (5.77)	10.64 (4.27)	144	0.751	0.03
	HF (%)	10.79 (5.73)	10.40 (7.53)	126	0.459	0.07
	LF:HF Ratio*	2.58 (1.77)	1.28 (1.64)	68	0.035	0.21
	LF (ms^2)	900.97 (1061.47)	1138.56 (3246.81)	100	0.177	0.14
	HF (ms^2)	432.30 (1214.74)	1262.12 (11197.00)	93	0.130	0.15
	Total Power (ms^2)	1344.12 (2117.44)	2266.99 (14689.89)	91	0.118	0.16
Nonlinear analyses	SD1 (ms)	12.61 (13.83)	19.81 (46.02)	96	0.148	0.15
	SD2 (ms)	26.55 (18.55)	32.30 (23.25)	87	0.098	0.17
	SD2:SD1	0.98 (0.57)	0.95 (0.82)	140	0.681	0.04
	ApEn	0.10 (0.07)	0.08 (0.09)	148	0.823	0.02
	SampEn	0.26 (0.10)	0.24 (0.17)	154	0.934	0.01
	Alpha 1	0.19 (0.08)	0.20 (0.19)	119	0.366	0.09
	Alpha 2	0.16 (0.04)	0.19 (0.07)	82	0.076	0.18
	D^2	1.06 (0.40)	1.21 (0.41)	112	0.285	0.11
	Mean line length	3.35 (3.67)	4.76 (46.07)	140	0.681	0.04
	Max line length	103.75 (33.40)	126.79 (81.50)	113	0.296	0.10
	Recurrence rate (%)	7.96 (3.20)	6.99 (8.15)	141	0.698	0.04
	Determinism (%)	1.06 (0.68)	1.04 (0.92)	156	0.972	0.00
	ShannonEn	0.29 (0.10)	0.29 (0.39)	125	0.445	0.08

^*^Results are statistically significant at the *p* < 0.05 level.

Results of groupwise variability in HRV parameters (Table 2) indicated a single statistically significant difference between groups with the LH:HF ratio of relative contribution to total spectral power. Cadets who endorsed clinically significant anxiety symptoms on the GAD-7 prior to their training during the CTP evidenced statistically significantly less variability over time in their relative power spectral density frequency band contributions, despite no statistically significant difference in averaged values over time (i.e., Standard deviation in LF:HF ratio over time, *p* < 0.05, *r* = 0.21; Average LF:HF ratio, *p* = 0.625, *r* = 0.05). Variability in the parasympathetic nervous system index during CTP was borderline significantly decreased in the positive screening group as well (i.e., PNS Index, *p* = 0.060). For the positive screening group, nonlinear analyses also indicated no statistically significant increases in SD2 and ALPHA 2 variability over time for Poincaré (*p* = 0.098) and Detrended Fluctuation Analyses, respectively, (*p* = 0.076). All differences were associated with small effect sizes.

## 4. Discussion

The current study was designed to examine variations in cardioautonomic lability among RCMP cadets based on positive or negative screens for one or more mental health disorders at the start of the CTP; however, only analyses regarding GAD were possible given that no cadets screened positive for clinically significant anxiety symptoms among the other included mental health disorders at the start of the CTP or those who did, did not participate in the Hexoskin data collection. Consistent with HRV literature in persons with anxiety disorders, we observed statistically significant decreases in HRV with small effect sizes (0.18–0.21) (Chalmers et al., 2014; Schmalenberger et al., 2019). Cadets who endorsed clinically significant anxiety symptoms on the GAD-7 at the start of CTP had a statistically significant decrease in the daily variability of the LF:HF ratio. Reduced LF:HF variability may better reflect a coupling of the sympathetic and parasympathetic contributions to HRV considering the mathematical and interpretive challenges when using the raw ratio value itself.

The LF:HF ratio has been widely used to compare vagal balance in biological signals such as heart rate, but the value remains is widely scrutinized based on interpretive variability within heterogenous clinical populations (Billman, 2013). The LF peak may represent sympathetic tone, whereas the HF peak may represent the parasympathetic tone, and the balance between the two represents the reciprocal interplay between the two competing but complementary pathways (Billman, 2013; Barbieri et al., 2017). There were no statistically significant groupwise differences in the variables of age, sex, or number of recordings available for analysis; nevertheless, there were differences in time varying latent neurobiological adaptations for acute or chronic shifts in the HPA axis associated with chronic stressors.

The evaluation of HRV in persons with anxiety disorders has been widely used, but there are considerable methodological discrepancies that limit clinical applicability and interpretation (Billman, 2013; Faes et al., 2013; Hayano and Yuda, 2019). In participants with GAD, few researchers have reported statistically significant decreases in HF spectral power and percent contribution to total spectral power (Lyonfields et al., 1995; Pittig et al., 2013), and others report no differences compared to age-and sex-matched healthy controls at rest (Hammel et al., 2011; Chalmers et al., 2014). Similar trends have been observed in those with panic disorder, with statistically significant decreases in HF percent contribution to total power spectral density compared to age-and sex-matched controls (Hammel et al., 2011; Fisher and Woodward, 2014; Chalmers et al., 2016). Decreases in HF spectral power and relative percent contribution to total power spectral density have been observed in participants with any anxiety-related disorder, regardless of specific diagnosis (Hedges’ *g* = −0.29, *p* < 0.001), all associated with small to moderate effect sizes (Chalmers et al., 2014).

The observably greater attentional bias towards threat perception in anxiety disorders has been documented to facilitate the maintenance of higher-than-normal levels of corticotropin releasing factor and higher basal levels of cortisol (Flandreau et al., 2012). Threat perception and HPA axis deviations lends perspective to the role that persistent stress can induce persistent latent neurobiological adaptations for neuroendocrine mediated changes in cardiac function (Edwards and Guilliams, 2010). The same neurobiological adaptations may support an acute hypersensitivity to threatening stimuli associated with activation of the HPA axis, and acutely decreased HRV in the HF band of the power spectral density function (Miu et al., 2009; Juruena et al., 2020; Spangler et al., 2021). The decrease in variability within the HF band is believed to be due to an increase in heart rate and a decrease in parasympathetic lability associated with decreased vagal inhibition of the sinoatrial node (Shinba, 2017; Spangler et al., 2021). The increased presence of circulating catecholamines support an increase in cardiac output in response to a threatening stimulus (Hammel et al., 2011; Pittig et al., 2013; Spangler et al., 2021). An increase in cardiac output affords a reduction in harmonic variability on a beat-to-beat basis as parasympathetic withdrawal is largely reported as the greatest contributing factor for rapid decreases in variability, while circulating catecholamines largely increase heart rate and decrease variability (Hagerman et al., 1996; Nichols et al., 2011; Barbieri et al., 2017; Chen et al., 2017; Owens et al., 2018).

HRV measures are designed to describe complex cardiovascular regulation mechanisms that coalesce from sympathetic and parasympathetic regulatory pathways, with near infinite variability (Task Force of the European Society of Cardiology and the North American Society of Pacing and Electrophysiology, 1996; Vila et al., 2019). The HPA axis deviates from rest in support of an autonomic balance that favors the conditional requirements of an acute stressor (DeBeck et al., 2010). Prolonged or repeated exposure to stressors, such as PPTEs, precipitate repeated HPA axis deviations and may cause maladaptive long-lasting effects to the normal stress response (Edwards and Guilliams, 2010; Flandreau et al., 2012; Juruena et al., 2020; Tafet and Nemeroff, 2020). Acute and chronic stressors are associated with decreases in autonomic lability (DeBeck et al., 2010; Shinba, 2017; Spangler et al., 2021); however, the mechanisms that are responsible for such changes vary in meaningful ways. An acute stressor (DeBeck et al., 2010), such as operational and organizational stressors, will precipitate a decrease in autonomic variability due to a situationally matched increase in heart rate and preferential shift in metabolism (Owens et al., 2018; Spangler et al., 2021). Maladaptation to a chronic stressor, such as repeated exposure to PPTEs, precipitates a decrease in autonomic variability due to exhausted or blunted autonomic responses (Kawachi et al., 1995; Gorman and Sloan, 2000; Hammel et al., 2011; Harrewijn et al., 2018). The current results support the coupling of the parasympathetic and sympathoinhibitory pathways within the medullary regions of the brainstem. The evidence supports the presence of varying degrees of HPA axis deviations observable at least in part by HRV metrics in a free living population (Pivatelli et al., 2012; Barbieri et al., 2017; Shinba, 2017; Chang et al., 2020).

The current study focused on the HRV parameters of RCMP cadets who endorsed clinically significant anxiety symptoms on the GAD-7 at the start of the CTP compared to the age-and sex-matched peers. The results were consistent with research evidencing the association between GAD and increased and prolonged activation of the HPA axis (Juruena et al., 2020). Persons with GAD experience excessive worry and anxiety related to several topics (e.g., money, health, family; Pelletier et al., 2017). The anxiety and worries are difficult to control, are experienced nearly every day for at least 6 months, and cause significant distress or impairment to daily activities (Pelletier et al., 2017). The chronic nature of GAD helps explain the association with clinically significant symptoms and decreased HRV (Kessler et al., 2001). Individuals who develop GAD in adolescence or early adulthood often remain undiagnosed for 10 years (Kessler et al., 2001). The extended lapse between the onset of GAD symptoms and diagnosis is concerning when the delay impedes delivery of the appropriate evidence-based treatment. Screening for GAD in early career PSP is warranted as earlier identification and intervention could improve both their mental and physical health. Information regarding when participants in the current study started experiencing symptoms of GAD was not available at the time of analyses; thus, the current sample may have heterogeneity regarding their autonomic lability due to time varying adaptations to acute or chronic stressors. Future researchers should include serial observations of all cadets as they begin their careers as PSP to observe acute (i.e., daily occupational stressors) and chronic stressor exposure (i.e., exposure to PPTEs) to evaluate the potential for recognizable changes in autonomic lability associated with autonomic maladaptation.

### 4.1. Strengths and limitations

A significant strength of our study is the observation of HRV over a controlled timespan, and the implementation of volitional participation during CTP. The CTP provides a unique and predictable environment, with the ability to screen cadets prior to training and evaluate a plethora of biometric signals during their time during training and thereafter. The CTP provides novel information that we can use to build injury models, and investigate the existence of, or observe the development of, neurobiological adaptations to stress that can manifest as PTSI. The same neurobiological adaptations present a unique perspective on PTSI as we can longitudinally evaluate cadets based on their anxiety symptoms as measured by the GAD-7 questionnaire and observe groupwise differences between acute and chronic PTSI. Groupwise and longitudinal analyses can provide unique insights about the neurobiological adaptations that may occur due to trauma exposure prior to their training, or while operating as PSP.

The study limitations include poor ECG recording quality for many records collected, changes in volitional participation over time precipitating non-equidistant sampling intervals, participant sociodemographic heterogeneity, and no reporting of if or when cadets may have been clinically diagnosed with anxiety disorders or the duration of their anxiety symptoms. Additionally, the absence of positive mental health disorder screens other than the endorsement of clinically significant anxiety symptoms on the GAD-7 limits the generalizability of the results, given the low rates of mental health disorder prevalence in all cadets at the start of training and the characteristics of volitional participation while wearing the Hexoskin garments. Left censorship bias is a particular challenge due to the often-heterogenous presentation of acute but appropriate stress responses, compared to long term neurobiological maladaptation that can precipitate from chronic stressor exposure. The small number of cadets who screened positive for clinically significant anxiety symptoms on the GAD-7also limits the generalizability of the results, although future analyses will examine longitudinal trends with an expanded sample as data collection continues in the years to come.

## 5. Conclusion

The role of stress and anxiety on HPA axis deviations as evaluated by HRV has been extensively studied, although with mixed and heterogenous results. Our study was designed to examine differences in cardioautonomic regulatory pathway integration in cadets that screened positive for GAD prior to their training at CTP, compared to age-and sex-matched community controls. Our results support the current literature, with statistically significant differences between groups, suggesting that cadets with clinically significant anxiety symptoms on the GAD-7 prior to CTP show less variability in the LF:HF ratio over the course of their 26-week training program compared to cadets who did not endorse symptoms. The results have important implications for future investigations of cardioautonomic dysfunction and chronic HPA axis deviations in persons with anxiety disorders, suggesting a statistically significant decrease in serial recording variability, rather than a statistically significant decrease in raw values, when observed during the CTP.

## Data availability statement

The datasets presented in this article are not readily available because data access will not be provided due to the sensitive nature of the content. Requests to access the datasets should be directed to jonathan.burry@uregina.ca.

## Ethics statement

The studies involving humans were approved by University of Regina Institutional Ethics Review Board. The studies were conducted in accordance with the local legislation and institutional requirements. The participants provided their written informed consent to participate in this study.

## Author contributions

RNC, TT, KM, RS, JN, and KA: conceptualization. RNC, TT, RS, TA, GA, GK, LL, KM, LJ, JN, and KA: methodology. RNC, TT, RS, GK, KM, and LJ: validation. RNC, TT, RS, KM, and LJ: formal analysis. RNC, GK, JPN, and SS-Z: investigation. RNC, RK, and SS-Z: resources. RNC, TT, GK, and JPN: data curation. TT, RNC, KM, LJ, JN, and KA: writing – original draft preparation. TT, JPN, KA, KM, LJ, JN, RS, TA, SS-Z, LL, RK, GA, GK, and RNC: writing – review and editing. All authors viewed and approved the submitted version of the manuscript.
